# Investigations on the Fatigue Behavior of 3D-Printed and Thermoformed Polylactic Acid Wrist–Hand Orthoses

**DOI:** 10.3390/polym15122737

**Published:** 2023-06-19

**Authors:** Diana Popescu, Florin Baciu, Daniel Vlăsceanu, Rodica Marinescu, Dan Lăptoiu

**Affiliations:** 1 Department of Robotics and Production Systems, Faculty of Industrial Engineering and Robotics, University Politehnica of Bucharest, 060042 Bucharest, Romania; diana.popescu@upb.ro; 2 Department of Strength of Materials, Faculty of Industrial Engineering and Robotics, University Politehnica of Bucharest, 060042 Bucharest, Romania; florin.baciu@upb.ro; 3 Department of Orthopedics, Carol Davila University of Medicine and Pharmacy, 050474 Bucharest, Romania; rodica.marinescu@umfcd.ro; 4 Department of Orthopedics, Colentina Clinical Hospital, 020125 Bucharest, Romania

**Keywords:** 3D printing, thermoforming, wrist–hand orthosis, mechanical behavior, customization, flexural fatigue, 3D Printing Point-of-Care

## Abstract

Additively manufactured wrist–hand orthoses (3DP-WHOs) offer several advantages over traditional splints and casts, but their development based on a patient’s 3D scans currently requires advanced engineering skills, while also recording long manufacturing times as they are commonly built in a vertical position. A proposed alternative involves 3D printing the orthoses as a flat model base and then thermoforming them to fit the patient’s forearm. This manufacturing approach is faster, cost-effective and allows easier integration of flexible sensors as an example. However, it is unknown whether these flat-shaped 3DP-WHOs offer similar mechanical resistance as the 3D-printed hand-shaped orthoses, with a lack of research in this area being revealed by the literature review. To evaluate the mechanical properties of 3DP-WHOs produced using the two approaches, three-point bending tests and flexural fatigue tests were conducted. The results showed that both types of orthoses had similar stiffness up to 50 N, but the vertically built orthoses failed at a maximum load of 120 N, while the thermoformed orthoses could withstand up to 300 N with no damages observed. The integrity of the thermoformed orthoses was maintained after 2000 cycles at 0.5 Hz and ±2.5 mm displacement. It was observed that the minimum force occurring during fatigue tests was approximately −95 N. After 1100–1200 cycles, it reached −110 N and remained constant. The outcomes of this study are expected to enhance the trust that hand therapists, orthopedists, and patients have in using thermoformable 3DP-WHOs.

## 1. Introduction

The rate of deployment of Additive Manufacturing (AM) applications in healthcare is continuously increasing alongside the research and scientific literature in the field [1,2]. This is explainable as the AM technology favors customization and design freedom, hence addressing patients’ anatomical characteristics and individual needs [3]. Moreover, it enables delocalized manufacturing and a simplified supply chain [4], thus allowing production within hospitals and medical centers—the implementations of 3D Printing Points-of-Care (3DP-POCs) being the current focus [5]. All of these advantages recommend 3DP (the colloquial name for the AM technology, usually also designating the process based on material extrusion—MEX) for the orthotics domain, as a modern manufacturing solution replacing the traditional methods of manually producing upper- and lower-limb static orthoses (splints and casts) [6,7].

Tendonitis, sprains and strains, overuse syndrome, contusions, spasticity or muscle weakness, arthritis, wrist instabilities and fractures [8,9] are among the medical conditions that call for immobilization by splints or casts for short periods of time (days, weeks) or permanently in the case of chronic conditions [6,10]. Therefore, besides functionality, it is mandatory to also consider the patient’s comfort and adherence to wear. In this regard, the literature covers the advantages of 3DP-WHOs over traditional fiberglass, thermoplastic or plaster orthoses, such as coolness, lighter weight, recyclable material, less skin irritation and itching, better hygiene, less perspiration and odor reduction [7,11,12], easy and rapid placement and a good fit to the patient limb, while satisfying the clinical requirements of maintaining immobilization [13].

However, despite the benefits and the large availability of MEX equipment (3D printers) and feedstock, the use of 3DP-WHOs is not common in clinical practice. Two recent systematic reviews addressing this field [6,14] evidenced a very limited number of level II studies, randomized and prospective clinical trials [8,9,15] aimed at assessing the efficiency of 3DP-WHOs in comparison to traditional orthoses by using functional criteria and patient feedback (VAS—Visual Analog Scale, QUEST rating—Quebec User Evaluation of Satisfaction with Assistive Technology, JTHFT—Jebsen–Taylor Hand Function Test, PRWE—Patient-Rated Wrist Evaluation). The majority of the studies reviewed in [6,14] are case reports, case series or retrospective case series, all showing good functional outcomes, and patients favoring 3DP-WHOs. At the same time, these studies note the dependence of 3DP-WHOs’ production on engineering skills, dedicated 3D scanning equipment and software, as well as the long printing times (e.g., 18–20 h [16] or 6 h in [9]). These aspects hamper 3DP-WHOs’ clinical use as most hospitals or healthcare centers lack reverse engineering equipment, dedicated 3D modeling software or 24/7 designer support. In this sense, solutions were proposed to semi-automate the design process [17,18,19]. There is also the flat-shaped orthoses approach [20,21] that offers an alternative to the long manufacturing time and to the patient 3D data acquisition and processing. Moreover, the 3D modeling process of the patient-tailored orthoses can be replaced with a web-based app that uses a parametric design customized according to the patient anatomic dimensions (see Section 1.1).

Equally as important as implementing a fast and efficient manner of producing bespoke 3DP-WHOs is proving their mechanical resistance during tasks specific to daily activities, thus gaining the confidence of orthopedists, hand therapists and patients. In this sense, the literature review conducted in Section 1.2 made clear the scarcity of studies investigating 3DP-WHOs’ mechanical behavior, while the flat 3D-printed and then thermoformed WHOs have never been examined from this standpoint. The current paper focuses on this niche by comparing the mechanical performance of the 3DP-WHOs built in the ready-to-use form (as typical for the 3D scanning or CT/MRI based approaches [22], denoted further as 3DP-WHO1, with the mechanical performance of the flat-printed WHOs, denoted further as 3DP-WHO2, thermoformed to fit the patient hand [21]). Flat-shaped WHOs are easier and faster to produce, and, if necessary, allow the embedding of sensors or therapeutic magnets; the research question to answer is whether they provide a similar mechanical resistance as the orthoses directly 3D-printed in the hand-shaped form. In this study, the 3DP-WHOs were subjected to three-point bending tests corresponding to wrist flexion/extension movements, and comparatively analyzed. Moreover, the durability of thermoformed 3DP-WHOs was assessed by conducting fatigue bending tests.

### 1.1. 3DP-WHOs’ Development Approaches

Any AM process requires the existence of the digital 3D model of the object to be manufactured. In AM-based medical applications, ‘reverse engineering’ the patient to reconstruct his/her anatomy and then designing the orthoses, prostheses or surgical guides are the steps to follow for patient-tailoring the device or product [13,22]. Once the digital model is available, the suitable material and AM process are selected. AM based on MEX and a powder fusion process (SLS—Selective Laser Sintering) are preferred for orthoses manufacturing, with MEX having the advantages of equipment and feedstock affordability. Other AM processes are also reported in the literature, such as Polyjet, for producing multi-material orthoses, as detailed further below when discussing the literature review results. The most common materials for 3DP-WHOs include polylactic acid (PLA), acrylonitrile butadiene styrene (ABS), or polyamide 12 (PA12).

**Figure 1:**
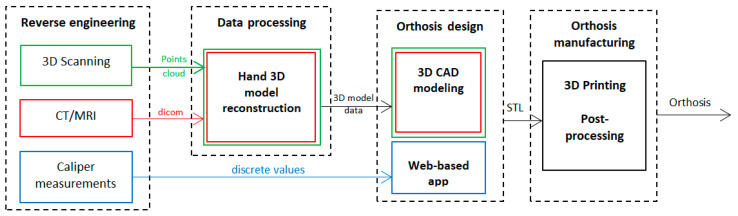
3DP-WHOs’ development flows.

Regarding the patient data acquisition, three approaches can be currently distinguished, with the type of equipment determining the subsequent development steps (Figure 1): 3D scanning-based; medical imaging-based (CT/MRI); caliper-based.

The first two approaches require 3D modeling and medical reconstruction knowledge, and dedicated software and equipment, while the third solution is based on the ability to correctly measure the patient hand and to thermoform the 3DP-WHOs for a good fit on the upper limb, skills which are common for hand specialists. Moreover, in the latter approach, the 3DP-WHO is built in a flat position [21], thus reducing the print time and cost in comparison to the hand-shaped orthoses 3D-printed usually in a vertical position that require support structures for openings or overhang features.

### 1.2. Literature Review

As mentioned in the introductory section, the recent reviews in the field present relevant information on the state of the art, including measured outcomes related to hand functions, functionality and patient satisfaction while wearing 3DP-WHOs [10], as well as the design approach and software, and production cost and time assessments [6]. However, so far, no data have been gathered on 3DP-WHOs testing for the evaluation of compliance with the mechanical performance criteria (such as bending, fatigue or impact). Therefore, a systematic search in the PubMed, Web of Science and Scopus electronic databases was performed by using the following keywords: (“3D printing” OR “additive manufacturing” OR “material extrusion” or “selective laser sintering”) AND (orthosis OR splint OR cast OR brace) AND (hand OR “upper limb” OR wrist) AND (test OR strength OR resistance OR bending OR fatigue OR “finite element” OR “mechanical behavior” OR strength). English language studies up to November 2022 were included. Title and abstract screening were performed by two authors after performing duplicates removal by using Mendeley Desktop 1.19.8 software. The exclusion criteria were the following: non-human studies, dynamic orthoses, spinal cord orthoses, occlusal splints, and studies related to casting manufacturing process. A total of 113 papers were initially selected, 36 papers being kept for a full-text read. Thirteen papers were identified as relevant in providing information on the mechanical behavior of 3DP-WHOs and/or on the use of finite element method (FEM) for investigating mechanical performance.

## 2. Materials and Methods

Two sets of orthoses were generated and manufactured (3DP-WHO1 and 3DP-WHO2) for this research, each one being typical for a development flow (Figure 1). They were tested by conducting three-point bending tests to investigate their resistance to wrist sagittal movements. Then, the thermoformed 3DP-WHO2s were subjected to flexural fatigue tests, which correspond to wrist flexion and extension. All 3DP-WHOs were made out of polylactic acid—PLA (Devil Design Sp. J., Mikołów, Poland). Acrylo butadiene styrene—ABS (Stratasys Inc., Eden Prairie, MN, USA)—was used for printing the 3DP-WHOs supports (Mojo 3D Printer, Stratasys Inc., Los Angeles, CA, USA) for the mechanical tests. For the fatigue tests, a bi-material forearm dummy was produced. The 3DP-WHOs and the mold for the dummy were manufactured on a Prusa Replica 3D printer (Prusa Research, Prague, CZ) using the Prusa Slicer 2.5.2 as slicing software.

### 2.1. Wrist–Hand Orthoses and Forearm Dummy

**Figure 2:**
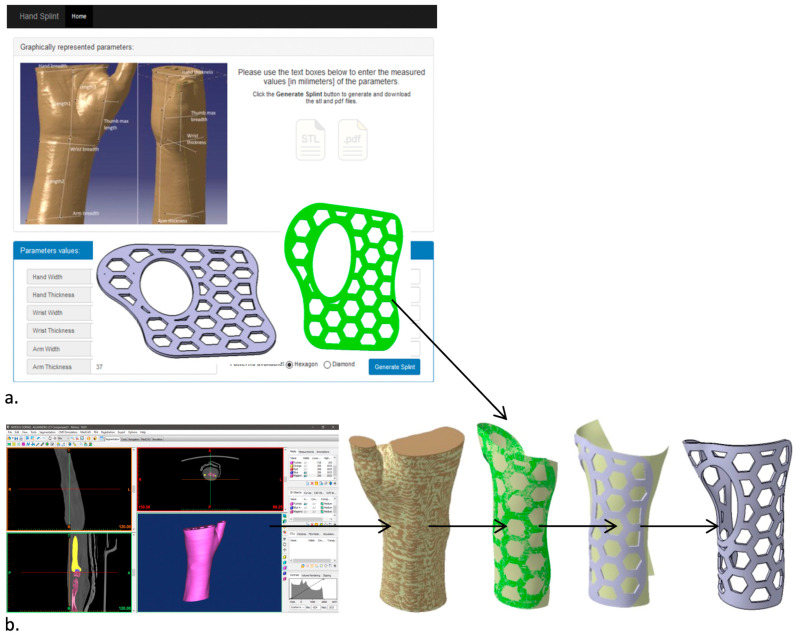
Orthoses design methodology and 3DP-WHOs’ development flows for (**a**) flat-printed 3DP-WHO2, and (**b**) hand-shaped 3DP-WHO1.

The 3DP-WHO2 was generated by using the app described in [21] and measuring the key dimensions of the wrist–hand of a male patient (Figure 2a), while the 3DP-WHO1 was based on the 3D hand model of the same person, reconstructed from the CT data (Mimics 10, Materialise NV, Leuven, Belgium). Their thickness was set to 2.3 mm. The 3DP-WHO1 and 3DP-WHO2 models included ventilation pockets with a hexagonal shape, resulting in a 35% weight reduction. A surface of the flat orthosis (the green surface in Figure 2a) was wrapped on the surface extracted from the 3D hand model (as presented in the flow in Figure 2b) by using the CATIA V5 Generative Surface Design workbench (Dassault Systemes, Velizy-Villacoublay, France). This method allowed us to generate similar orthoses in terms of surface area and volume, for a valid comparison.

**Figure 3:**
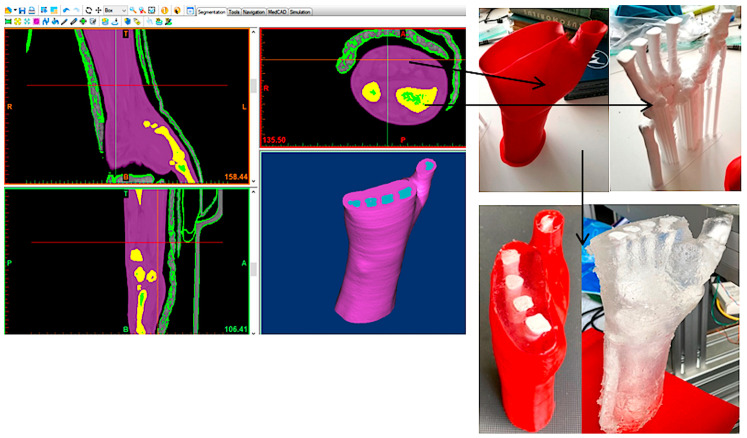
Forearm dummy production process.

After segmenting the medical imaging data for tissue, and adding a core composed of the forearm bones, candle gel (with a density of 912 kg/m^3^, similar to the density of the human upper limb soft tissue [23]) was cast into a 3D-printed mold. The development flow depicted in Figure 3 was based on the same CT data used for designing the 3DP-WHO1. To enable the removal of the support structures sustaining the fingers, the two STL files generated using Mimics were 3D-printed separately. The two prints (corresponding to the forearm bones and to the mold) were then assembled together. The bones were 3D-printed with 20% infill density and 5 shells (2 mm, i.e., approximately the cortical bone thickness), while the hand exterior consisted of two shells so that they can be easily cut away after the solidification of the gel, without top/bottom layers, and only brim adhesion support.

### 2.2. 3D Printing

The main 3DP process parameters are presented in Table 1, which also shows the build orientation and printing times for each WHO. To ensure comparability of the mechanical testing results, it was important to assure not only similar designs, but also similar weights for both types of orthoses. Achieving this required careful consideration of the process parameters’ settings. For the vertically printed orthoses, a high infill density, closer to 100%, was necessary due to the small layer thickness (2.3 mm) and the risk of fragility within the interior structure/infill lay between the two perimeters. Furthermore, the orthoses’ height (160 mm) increased the likelihood of detachment from the printing platform in case of a smaller density value because of lack of stiffness, as resulted from previous experience. To achieve similar weights (in this case 33 g), we experimented with various combinations of (integer) infill densities for both the vertical and flat orthoses. These experiments were conducted using Ultimaker Cura 4.8.0 (Ultimaker BV, Geldermalsen, NL) simulations. Through this iterative process, infill density values were determined (97% infill density for the vertical orthosis and 40% infill density for the flat orthosis) that resulted in comparable weights for the orthoses. The fact that the infill density (and thus the weight) can be reduced when printing the orthoses as flat, while maintaining the required stiffness as proved by the results of the mechanical tests conducted in this research, is another advantage of our approach.

**Figure 4:**
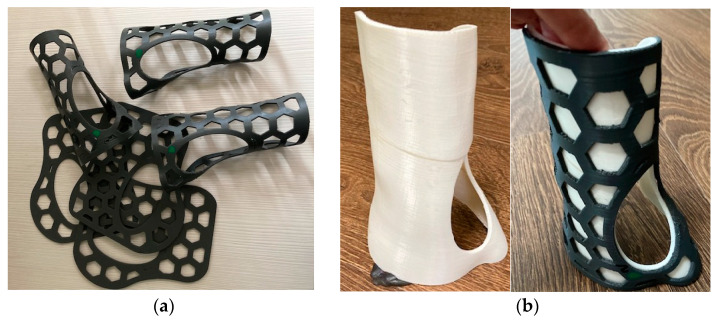
(**a**) Flat and hand-shaped 3DP-WHOs with hexagonal pockets for three-point bending tests; (**b**) mold and thermoformed 3DP-WHO2.

Afterwards, the 3DP-WHO2s (Figure 4a) were thermoformed in warm water (at 85 °C) and shaped in the form of the hand by using a 3D-printed mold made out of ABS (Figure 4b).

### 2.3. Experimental Tests

**Figure 5:**
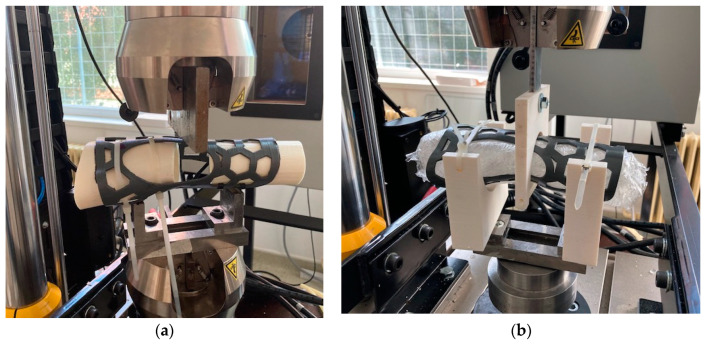
(**a**) Experimental set-ups: 3-point bending tests; (**b**) 3-point fatigue bending.

To assess the strength of 3DP-WHOs in wrist sagittal movements, a three-point bending test method was chosen. The orthoses were installed on two supports (in the forearm zone and in the palm/fingers zone) with self-locking nylon cable zip ties, leaving the wrist zone unsupported (Figure 5a). Attention was paid to ensure a similar tightening for all WHOs by measuring the free-end length of the zip ties. The load was applied on the orthoses in the wrist region, with the deflection points being separated at 100 mm.

The Instron 8872 Universal Test Machine (Instron Inc., Norwood, MA, USA) was used for the mechanical tests. Three samples of 3DP-WHO1 and three samples of 3DP-WHO2 were subjected to three-point bending tests, and the results were comparatively analyzed. Moreover, 3DP-WHO2s were subjected to a fatigue bending test using tension–compression cyclic loads (Figure 5b) and the dummy forearm (presented in Section 2.1) as the interior support. Each sample underwent 2000 cycles (equivalent to an active wearing of the splint for around three–four weeks [24]) at 0.5 Hz frequency and ± 2.5 mm displacements (flexion–extension).

## 3. Results and Discussion

### 3.1. Literature Survey Outcomes

Table 2 shows the most relevant data extracted from the full-text reading of the papers. It should be mentioned that only four papers reported the outcomes of experimental investigations on the mechanical properties of 3DP-WHOs. For the mechanical tests, one study [25] used a cadaver forearm, whereas in the other investigations, the 3DP-WHOs were mounted on specially designed supports inside the testing apparatus. A TPU (thermoplastic polyurethane) model of the entire forearm was used in one study as an internal support for the orthoses during the tests [26]. Most of the reviewed studies considered three-point bending tests for simulating the wrist flexion, although Cazon et al. [27] and Hoogervorst et al. [25] focused on all hand movements (radial, ulnar, and flexion).

With the exceptions of Chen Y et al.’s research in which CT imaging was used [28], and Sorimpuk et al. who used a flat model of the orthosis [20], in all the other studies, the 3DP-WHOs are designed starting from 3D scans of the patient hand. Methods to semi-automate the WHO design process were considered in many papers (Table 1) as solutions to decrease the design time and dependence on 3D modeling skills. In the same context of the design and manufacturing time reduction, Kim et al. proposed an interesting solution to make WHOs from two parts from which only the interior frame is patient-customized, while the outer frame is injection-molded and made on sizes corresponding to Korean population forearm data [29]. Long printing times are mentioned as a drawback in the majority of reviewed studies.

Sorimpuk et al.’s study was the only one found on the topic of flat 3DP-WHO mechanical behavior. However, the orthosis model is generated based on the average dimensions of adult Malaysians’ hands [20] and it is not patient-customized, while the flat model of WHO was digitally wrapped on the forearm 3D model for performing FEA. No experimental tests are presented for validating the simulation model.

In twelve papers, FEM is used for investigating the mechanical behavior of 3DP-WHOs under different loads occurring in daily use, including simulations of accidental impact [30,31,32] and thermal analysis [33]. FEM is also used for the topological optimization of 3DP-WHOs’ design [18,29]. The reported limitations of the numerical simulations relate mainly to the anisotropic characteristics of 3D prints. The results of the FE-based simulations performed on orthoses with different dimensions, materials and designs proved that 3DP-WHOs are suitable in terms of mechanical resistance and can provide the required immobilization (i.e., the displacement analysis under loads).

In summary, the literature review reveals the following key findings:

- Out of thirteen papers, four conducted experimental assessments on the mechanical behavior of 3D-printed wrist–hand orthoses (3DP-WHOs);
- No research has been conducted to compare the mechanical behavior of 3DP-WHOs manufactured in hand-shaped form with thermoformed 3D-printed orthoses;
- The most commonly used mechanical test for 3DP-WHOs is three-point bending, specifically for wrist flexion movement;
- Six papers focused on discussing WHO production through the MEX process, while the remaining papers discussed the utilization of SLS, SLA and Polyjet processes;
- For MEX orthoses, the following materials were used: PLA, ABS, PA12, HIPS;
- In two studies, multi-materials or hybrid manufacturing were used for producing the orthoses;
- For the purpose of reverse engineering the patient hand and designing the hand-shaped orthoses, eleven out of thirteen papers performed 3D laser scanning and associated data processing.

**Table 2:** Summary of the studies on 3DP-WHOs’ mechanical testing and FE simulations.

Study	Acquisition Method	Manufacturing Process/Material	Mechanical Testing	FEA	Observations
	WHO Design Method/Software				
Agudelo-Ardila, et al., 2019 [33]	3D scanning, 3D handheld scanner	SLS process, ProX SLS 500 printer (3D Systems, Inc., Rock Hill, SC, USA, DuraForm ProX PA	No	Stress (load: 60N bending), thermal analysis (40 °C).	Comparison of manufacturing times for 3DP-WHOs vs. conventional WHOs.
	Meshmixer, 2 parts WHO, Voronoi structure, 2.5 mm thickness				
Buonamici, et al., 2019 [18]	3D scanning using a dedicated, in-house developed device	MEX process, Stratasys F370 (Stratasys Inc., Eden Prairie, MN, USA)—FDM printer, ABS M30	No	FEA used for topology optimization for reducing weight by ventilation area.	Child WHO printing time 7h 21min; male adult 18h 6min; 52 min modeling time.
	Customized semi-automatic design software based on Siemens NX 10; 2 parts WHO with zip ties; different designs for the ventilation holes (circular holes, Voronoi, topology optimization)				
Cazon, et al., 2017 [27]	3D scanning, ZScanner 800 3D laser scanner	Polyjet process, Object Connex printer (Stratasys Inc., Eden Prairie, MN, USA)—multi-material WHO: VeroWhitePlus, TangoBlackPlus	Yes, dedicated support for WHO testing, mechanical tests for radial, ulnar, flexion and extension movements	FEA using Creo 3.0, 4 hand movements: radial, ulnar, flexion and extension, torques (Vanwearingen torques used as reference: 14.8 Nm, 8.4 Nm, 11.4 Nm, and respectively 9.9 Nm) and loads on *x* and *z* directions.	Mechanical tests showed displacements of 3.46 mm, 0.97 mm, 3.53 mm, and 2.51 mm for flexors, extensors, radial deviators and ulnar deviators. In ulnar direction, 3DP-WHO had a greater displacement, as resulted in FEA. 3DP-WHO proved suitable for everyday use.
	Geomagic Studio 2013				
Chen, C.D. et al., 2019 [15]	3D scanning, Creaform Go!Scan50 scanner	MEX process, PLA	No	ANSYS, 3 parameters with 2 design values (WHO thickness, ventilation holes diameter, holes center distance); -flexion 30 N; extension 25 N; radial deviation 30 N; ulnar deviation 30 N -impact with 40 mm diameter steel ball of 0.3768 kg.	Material properties used in FEA were experimentally determined on specimens–ASTM D638, ISO 180.
	2 parts WHO tied with Velcro strips, designed using in-house solutions				
Chen, Y, et al., 2020 [28]	CT, Mimics 10.01 for forearm reconstruction	SLS process, EOS P395 (EOS GmbH, Krailling, Germany), PA2200	No	ANSYS Workbench 18; 6 loading conditions including anterior to posterior (AP), posterior to anterior (PA), medial to lateral (ML), lateral to medial (LM), inward (IR), and outward OR to calculate the displacement and stress; 400 N compression load on the palm along AP, PA, ML, LM; 1 Nm rotation moment toward the IR and OR of the palm, applied to the top end side of cast.	FE model for bone, soft tissue and cast. Immobilization using 3DP-WHO was effective. A total of 60 patients, 20 used 3DP-WHOs, patients satisfaction assessment. Long manufacturing times, not suitable for emergency situations.
	Solidworks 2015; 2 parts WHO				
Gorski, et al., 2020 [26]	3D scanning, David SLS-3 optical scanner	MEX process, Raise 3D Pro machine (Raise 3D Technologies, Nantong, China); ABS, PLA; PA12, HIPS; different infills, layer thicknesses; vertical and horizontal build orientation	Yes, three-point bending using a TPU phantom of the forearm	No	Different assessment criteria: manufacturing time and cost, strength dependence on process parameters and material, the patient wore the 3DP-WHOs for 15 min for yes/no comfort feedback.
	In-house dedicated design app (AutoMedPrint), 2 parts WHOs with snap fit connection, 4 mm thickness				
Hoogervorst, et al., 2019 [25]	3D scanning, 3D Structure Sensor infrared scanner	MJF process, HP Multi Jet Fusion Printer (HP Inc., Palo Alto, CA, USA), PA12	Yes, using a cadaver forearm; -flexion and extension of digits (1000 loading cycles (20–100 N tensile force), -pronation and supination of the hand (1000 cycles of torque (−0.5 to 0.5 Nm)), -three-point bending (1000 cycles (50–500 N))	No	Cadaver biomechanical study for assessing the stabilizing properties of 3DP-WHOs in comparison with traditional fiberglass cast. Only three-point bending results were statistically different, but with a very small value of the absolute motion: 0.44 (±0.48)mm.
	Open lattice design				
Kim & Jeong, 2015 [29]	3D scanning	Hybrid manufacturing PolyJet, Objet500 Connex (Stratasys Inc., Eden Prairie, MN, USA), ABS, (inner frame, 2 part) and injection molding (outer cover, 2 parts, PC)	No	ANSYS 13, FEA for determining the thickness of WHO outer cover subjected to 200 N impact force	Manufacturing time and cost reduction by customizing just the inner frame and its connection bumps, while the outer frame is available on sizes
	3D CAD software for inner frame, outer frame designed based on population forearm measurements data				
Li & Tanaka, 2018 [31]	3D scanning, Sense handheld 3D scanner	MEX process, Qidi Tech 1 3D printer (Qidi Tech, Ruian, China), ABS	No	FEA using Fusion 360 software; 30 N loads on the distal edge of the splint and lattice-structure area along three directions for simulating possible hits and stresses.	3DP-WHO tested on 10 healthy subjects, no significant discomfort reported (3 reports of itching)
	Semi-automated design using in-house app based on Rhinoceros 5 and Grasshopper 3D; 2–3 parts WHO connected with M3 screws, lattice structure				
Lin, et al., 2016 [32]	3D scanning, Artec Eva and Artec Space Spider 3D scanners	SLA process, RS6000 3D printer (UnionTech, Beijing, China), PP	No	FEA using ANSYS for strength assessment, 3MPa impact pressure applied on the ventilation holes zones.	-
	In-house modeling app for semi-automating the design process, 2 parts WHO, 2 mm thickness				
Lukaszewski et al., 2020 [34]	3D scanning	MEX process, FlashForge Creator Pro (Zhejiang Flashforge 3D Technology Co., Ltd., Jinhua, China); ABS; 5 types of samples in different build orientations, 15% infill density, linear pattern, 2 shells	Yes, three-point bending parts for: specimen, part of WHO, part of WHO with ventilation holes, full WHO	FEA using ABAQUS for calculating WHO modulus of elasticity, 100 N loads in the middle of the WHO placed in a horizontal position.	The WHO parts with ventilation holes built in horizontal plane have higher stiffness than those built vertically.
	WHO of 4mm thickness				
Modi, et al., 2020 [30]	3D scanning, HandySCAN 3D laser scanner	SLS process, EOSINT P395 (EOS GmbH, Krailling, Germany), PA 2200	No	FEA using Fusion 360; 100 N on the forearm area near proximal end of splint (as in impact), 30 N loads at the distal end of the splint near fingers as in accidental fingers bending.	The mechanical properties used in FEA were experimentally determined using specimens. Long development process (19 h).
	Design process using Meshlab, GeoMagic Studio and CATIA V5				
Sorimpuk, et al., 2022 [20]	Average dimensions of the forearms and hand circumference of Malaysian adults applied to a hand model from GrabCAD	MEX process, PLA (Ultimaker BV, Geldermalsen, NL)	No	FEA using Inventor 2017; 400 N load in X and Z directions of the cast, and 1 Nm bending moment along the Y direction of the cast.	FEA was performed on the digitally wrapped model of the 3DP-WHO. Results were compared with plaster traditional casts and SLS manufactured cast [28]. Reported printing time 3h 15 min. No process parameters details. No comparison with real 3DP-WHO.
	Flat-designed WHO with different ventilation pockets and specific adaptive pattern for the wrist joint curvature				

### 3.2. Results of Mechanical Tests

**Figure 6:**
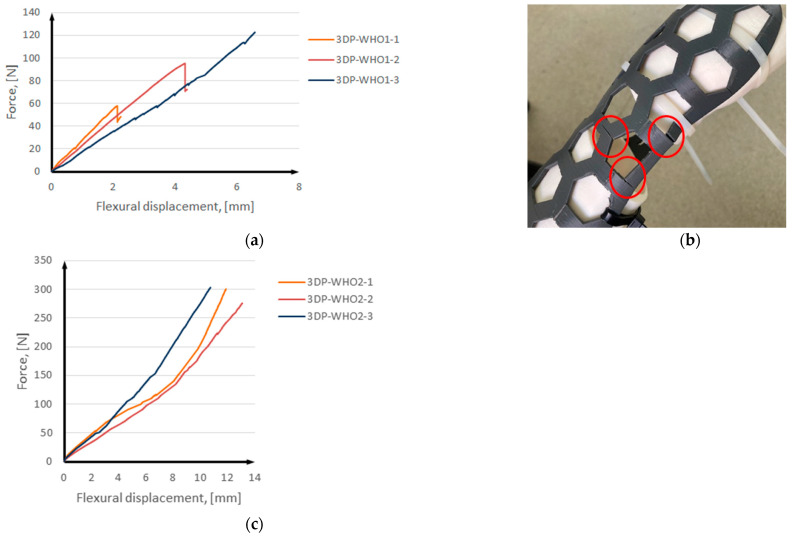
Results of three-point bending tests: (**a**) 3DP–WHO1s; (**b**) 3DP–WHO1 failure; (**c**) 3DP–WHO2s.

The results of the three-point bending tests are presented in Figure 6, with the mean force and displacement values listed in Table 3. The force–displacement data indicate that the 3DP-WHOs exhibit similar mechanical stiffness, with almost equally steep curves of up to around 50 N (Figure 6a,c). In cases where greater stiffness (i.e., immobilization strength) is necessary based on medical diagnosis, increasing the thickness of the thermoformable orthoses may be considered.

The 3DP-WHO1s samples failed around 60 N, 90 N, and, respectively, 120 N. Inter-layer fractures occurred in all cases (Figure 6b), which was expected considering the vertical build orientation and load direction. The presence of inter-layer defects can explain the differences in the load to failure. It should be noted that printing defects (lack of layer adhesion or inter-layer voids) are more common on tall parts with thin walls [35], as is the case of 3DP-WHO1s. These defects and the fact that the load is applied along the layers leads to the reduction of the structural integrity of these orthoses, thus reducing their life span or use at low loads (<100 N equivalent to 10 Kg). The samples were tested until cracks appeared (Figure 6b).

The experimental tests for 3DP-WHO2s were stopped at an applied load of 300 N (Figure 6c), which was considered as exceeding the typical value for this type of medical device according to the practice and literature data (Table 2). No material changes or breakage was noticed for any 3DP-WHO2. It was observed that the 3DP-WHO2s deformed in the elastic area, with no cracks being visible, and after removing the load, they returned to the original shape.

Cazon et al. comparatively assessed the conventionally manufactured WHOs and multi-material 3D-printed WHOs (obtained by using the Polyjet process), showing that 3DP-WHOs were more rigid in flexion, extension and radial directions [27]. The applied forces corresponded to 8% and 50% of a healthy person load, i.e., 53.7 N flexor, 41.4 N extensor, 56.4 N radial and 48.9 N ulnar for the 50% load, equivalent to the maximum strength of wrist rheumatoid arthritis [27,36]. Chen CD et al. developed an FE model of a PLA 3DP-WHO and applied loads of 30 N for flexion and 25 N for extension [15], but no mechanical tests were performed to validate this model. All the 3DP-WHO samples tested in our study withstand these loads. However, the measured displacements could not be related to the literature data as all the surveyed experimental studies considered full casts and not splints. Moreover, these casts had different pocket designs, masses and larger thicknesses (3 mm in [27], 4 mm in [26]), which would also make the comparison incorrect. Gorski et al. [26] evaluated the mechanical behavior of PLA, nylon, ABS and HIPS (high impact polystyrene) casts, 3D-printed using different process parameters. These WHOs were subjected to 300 N load and the experimental outcomes showed that none of them failed below 750 N [26].

Lukaszewski et al. applied a load of 100 N in the middle of a PLA wrist–hand cast (mass of 49 g) in a three-point bending test [34]. Tests were conducted on different models, and differences in the modulus of elasticity were observed as depending on the samples’ build orientation, but also on the presence or lack of ventilation pockets.

**Figure 7:**
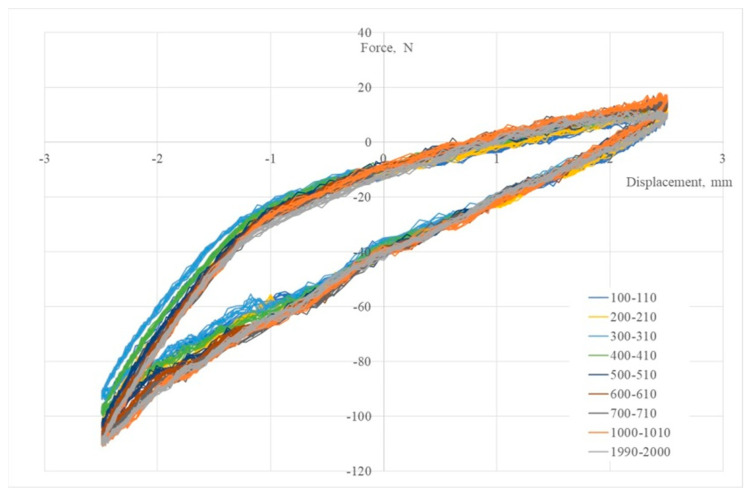
Hysteresis curves in bending fatigue tests for 3DP–WHO2.

**Figure 8:**
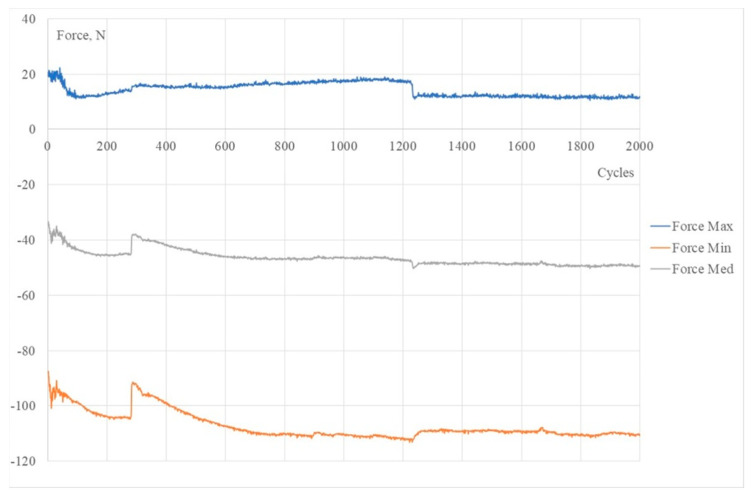
Force-cycle variation for a 3DP–WHO2 sample.

Figure 7 shows 3DP-WHO2 samples’ cyclic response when subjected to maximum-to-minimal load under different strain values. It can be noted that the hysteresis loops’ stabilization took place after 1000 cycles, the loops’ shapes being typical for viscoelastic materials. The applied load cycles did not cause the failure of any tested sample. The minimum force occurring during the flexural fatigue test was approximately −95 N, and after approximately 1100–1200 cycles, it reached −110 N and remained constant until the end (Figure 8). A possible explanation for this behavior might be the strain hardening, with more investigations being required in this regard. Additionally, in further research, it will be important to understand the effect of the orthoses’ design (thickness, ventilation pockets’ shapes and dimensions) on the fatigue limit, as criteria to consider in the design process based on the functional conditions of this medical device. Furthermore, studying the influence of the stress frequency on the fatigue behavior can provide useful data on 3DP-WHOs’ durability.

The flexural fatigue behavior of 3D-printed PLA specimens was previously studied for identifying the impact of process parameters on the fatigue strength [37,38]. However, only one reference was found on this type of mechanical test for WHO. A polyamide 3DP-WHO in the form of a cast encasing the forearm was investigated [25] for 1000 cycles between 50–500 N, at a frequency of 0.5 Hz. In their study, a cadaver arm was used, and the displacements were assessed with radiographs.

Both types of tests conducted in this research showed not only that the thermoformable 3DP-WHOs have a better quasi-static flexural behavior compared to 3D-printed orthoses vertically built in their almost ready-to-use shape (support structures’ removal being required as well), but they can also sustain long cyclic loads (tension–compression) which might occur during wear.

### 3.3. Discussion on 3D-Printed PLA Orthoses’ Thermoforming

Thermoforming sheets from polymeric material is a common industrial practice to obtain a large range of objects with complex shapes by using mechanical force applied to heated material and using forming tools such as molds and dies. However, this process is not commonly adopted when it comes to 3D-printed thin parts made from polymeric materials, despite interesting potential uses [39,40].

**Figure 9:**
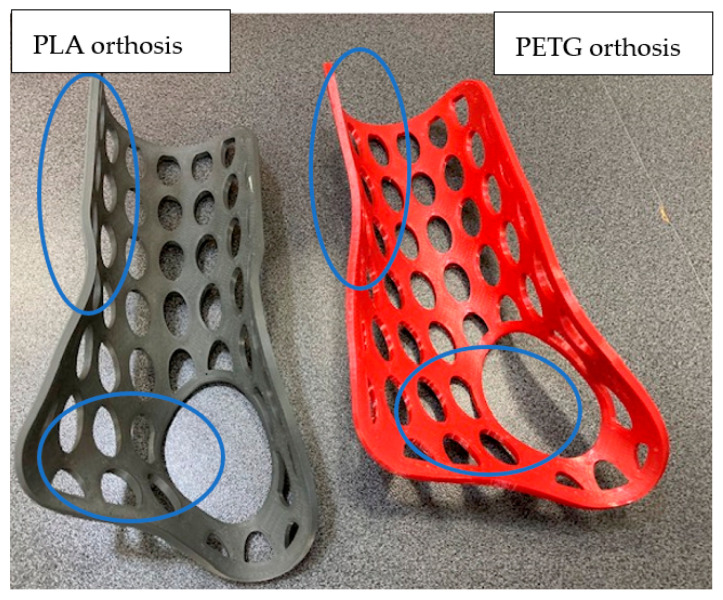
3D-printed cockup wrist–hand orthoses made from PLA (**left**) and PETG (**right**).

PLA is an aliphatic polyester made from renewable resources, such as sugar, corn, potatoes, and beets, with a glass transition temperature (Tg) ranging between 55 °C and 60 °C, meaning that it becomes soft and easy to deform at lower temperatures in comparison to other thermoplastics. PETG (Polyethylene Terephthalate Glycol-Modified—a thermoplastic polyester derived from petroleum-based sources), for example, exhibits a higher Tg of approximately 85 °C. Thermoforming a 3D-printed flat orthosis should take place at temperatures between Tg and Tcc (cold crystallization temperature of about 100 °C [41]), which allow keeping the material in a semi-solid state with sufficient molecular chain mobility while preventing unwanted crystallization prior to the forming process [42]. Since orthosis thermoforming occurs on the patient’s hand, temperatures that are too high can potentially cause burns, making it unsuitable for direct contact with the skin. Conversely, reducing the temperature to a level acceptable for the skin may result in challenges in achieving proper molding on the hand shape, particularly in the palm zone, as depicted in Figure 9 for the PETG orthosis. Within the marked zones in Figure 9, noticeable differences in deformations between the two material types can be observed, with the PETG orthosis failing to conform properly to the shapes of the forearm and palm. Wrapping the patient’s hand before thermoforming is advisable. In this research, PLA orthoses were heated at 80 °C, while PETG orthoses at 100 °C.

Thermoforming is possible as PLA has rubbery properties above Tg. The phases involved in thermoforming include heating, molding and cooling. Heating determines the molecular movement of the polymer chains facilitated by heat energy, which increases their mobility and decreases intermolecular tensions. Thus, the material becomes soft and ready to be molded, a phase in which the polymer chains align in the direction of the stretching force. Through the cooling process, PLA temperature is lowered below Tg, allowing the polymer chains to regain their rigidity, effectively solidifying the material in the desired shape. In the case of 3D-printed PLA thin objects, such as the orthoses, specific aspects related to infill pattern and infill density can occur, influencing the time required to reach the molted state for forming, as well as the cooling time. Attention should be paid to these characteristics, and they are the subject of further studies.

## 4. Conclusions and Further Work

In this research, the flexural performance of 3DP-WHOs manufactured by two approaches (i.e., customized upper-limb orthoses manufactured directly in a hand-shaped and flat-shaped form, and then thermoformed orthoses) was assessed in three-point quasi-static and uniaxial fatigue and three-point flexural tests corresponding to the wrist flexion/extension (sagittal) movements. No such investigation had been conducted so far, as the systematic review of the literature showed.

The rationale of this study was related to the observation that the flat-shaped 3DP-WHOs take less time for printing, cost less, and can be generated without necessitating 3D modeling skills. Therefore, they are easier to implement in 3DP-POCs. In this context, the issue of interest was whether their mechanical performance is also suitable for conditions typical for daily uses. The experimental outcomes indicated a positive answer to this research question. Moreover, 3D-printed and thermoformed WHOs proved resistant to cyclic flexural loading up to 2000 cycles, with the orthoses being placed on bi-material phantom aimed at mimicking the human hand during the tests.

Further research will be focused on understanding the dependency between the design, material, process parameters (infill-related, printing temperature, layer thickness), testing conditions, and the flexural strength of these orthoses to gather more data to support hand specialists’ decisions in prescribing these 3D-printed medical devices.

## Figures and Tables

**Table 1:** 3DP-WHO2 manufacturing information.

3DP-WHO1	3DP-WHO2	Process Parameters
Hand-tailored form–build orientation 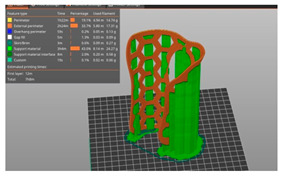	Flat form–build orientation 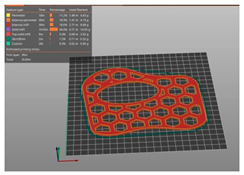	Printing temperature: 215 °C Bed temperature: 70 °C Infill pattern: grid 2 shells Top/bottom layers: 2 Infill density WHO1: 40% Infill density WHO2: 97%
Printing time: 7 h 8 min	Printing time: 2 h 39 min	

**Table 3:** Mean values of force and displacement for the tested samples.

Samples	Force at Break, [N]	Displacement at Break, [mm]
3DP-WHO1	81.13	4.41
3DP-WHO2	292.9	11.89

## Data Availability

Data available on request.
